# Two Needle Passes Achieve Similar Diagnostic Yield Compared to Three Passes Regarding Diagnosis of Solid Pancreatic Lesions in Endoscopic Ultrasound-Guided Fine Needle Aspiration

**DOI:** 10.3390/diagnostics11122272

**Published:** 2021-12-04

**Authors:** Eleni Koukoulioti, Georgios Tziatzios, Mario Tadic, Stavros Dimitriadis, Paraskevas Gkolfakis, Ekaterini Politi, Tajana Stoos-Veic, Petra Turcic, Alexandros Chatzidakis, Lazaros-Dimitrios Lazaridis, Maria Farmaki, Antonios Vezakis, Konstantinos Triantafyllou, Andreas Polydorou, Ioannis S. Papanikolaou

**Affiliations:** 1 Hepatogastroenterology Unit, Second Department of Internal Medicine–Propaedeutic, Research Institute and Diabetes Center, Medical School, National and Kapodistrian University of Athens, ‘‘Attikon” University General Hospital, 12462 Athens, Greece; e.koukoulioti@gmail.com (E.K.); g_tziatzios@yahoo.gr (G.T.); dimitriadiss@gmx.de (S.D.); alexandroshatzidakis@gmail.com (A.C.); dimlaza@hotmail.com (L.-D.L.); ktriant@med.uoa.gr (K.T.); 2 Endoscopy Unit, Department of Gastroenterology, Hepatology and Clinical Nutrition, Dubrava University Hospital, 10000 Zagreb, Croatia; mtadic1@gmail.com (M.T.); tajanaveic@hi.t-com.hr (T.S.-V.); 3 Department of Gastroenterology, Hepatopancreatology and Digestive Oncology, Erasme University Hospital, Université Libre de Bruxelles, 1070 Brussels, Belgium; pgkolfakis@med.uoa.gr; 4 Department of Medical Oncology, Institut Jules Bordet, 1070 Brussels, Belgium; 5 Department of Cytopathology, Aretaieion Hospital, Medical School, National and Kapodistrian University of Athens, 11528 Athens, Greece; ekpoliti@med.uoa.gr; 6 Department of Pharmacology, Faculty of Pharmacy and Biochemistry, University of Zagreb, Domagojeva 2, 10000 Zagreb, Croatia; petra.turcic@gmail.com; 7 2nd Department of Surgery, Aretaieion Hospital, Medical School, National and Kapodistrian University of Athens, 11528 Athens, Greece; mfarmaki19@gmail.com (M.F.); avezakis@hotmail.com (A.V.); apolyd@med.uoa.gr (A.P.)

**Keywords:** endoscopic, ultrasound, pancreatic, fine, needle, aspiration

## Abstract

Current guidelines advocate 3–4 passes with a fine-needle aspiration (FNA) to achieve high rates of diagnostic samples for malignancy when performing endoscopic ultrasound (EUS)-guided sampling of solid pancreatic lesions, in the absence of on-site cytologic evaluation. The aim of this study is to compare 2 vs. 3 needle passes in EUS-FNA for solid pancreatic lesions in terms of incremental diagnostic yield and to identify factors associated with the procedure’s outcome. In this retrospective study, 2 passes of EUS-FNA were found to have similar diagnostic yield compared to 3 passes for the diagnosis of solid pancreatic masses, suggesting that there might be no significant incremental tissue yield when 3 passes are performed.

## 1. Introduction

Endoscopic ultrasound-guided fine-needle aspiration (EUS-FNA) holds a pivotal role in the assessment of pancreatic masses given its ability to allow diagnostic tissue sampling in the majority (78–90%) of cases, along with a low adverse event rate [1,2,3]. Nonetheless, EUS-FNA remains a multistep diagnostic modality involving several factors (i.e., type and gauge of needles, use of suction, stylet) that can affect procedural outcomes, with the presence of an on-site cytopathologist (rapid on-site evaluation (ROSE)) being a cardinal one [4]. Despite its established efficacy, routine participation of a cytopathologist during EUS-FNA is not standard of care in everyday clinical practice across many institutions [5]. In light of these observations, recent European Society of Gastrointestinal Endoscopy (ESGE) guidelines recommend 3 to 4 needle passes to be performed during evaluation of pancreatic masses, to provide an adequate tissue sample [6]. Still, the optimal number of passes in order to achieve a satisfactory diagnostic yield—in the absence of ROSE—remains to be elucidated, especially when evidence suggests that even a single needle pass may be sufficient to establish cytological diagnosis [7,8,9,10]. Moreover, routinely performing additional passes in every pancreatic mass may not only prolong procedure time, but also pose a significant burden regarding the potential for adverse events, the need for additional aspiration needles per case, which translates into augmented costs, and most importantly, the need for repeat procedures due to non-diagnostic specimens [11]. In this context, the aim of the present study was to compare the diagnostic yield between 2 and 3 needle passes in EUS-FNA of solid pancreatic lesions and to identify potential factors that might influence obtaining a diagnostic tissue sample.

## 2. Materials and Methods

### 2.1. Study Design and Participating Centers

This was a dual center, retrospective cohort study carried out in 2 European academic tertiary care centers with extensive experience and special focus on EUS-FNA for solid pancreatic masses (Athens, Greece—Center 1 and Zagreb, Croatia—Center 2).

### 2.2. Inclusion and Exclusion Criteria

Consecutive patients referred to one of the participating centers for EUS-FNA for the evaluation of a solid pancreatic mass between January 2016 and October 2019 were considered eligible for inclusion. Patients had been initially diagnosed with a consistent lesion by ultrasound, computed tomography, or EUS. Exclusion criteria comprised: age < 18 years, performance of one or more than 3 passes, known former diagnosis of pancreatic malignancy and cases where data regarding the final diagnosis were not available.

### 2.3. EUS-FNA Procedure Performance

Patients received the standard preparation for EUS-FNA and were nil by mouth for 6 h prior to the procedure. All procedures were performed by two experienced endosonographers (each having completed ≥200 FNA procedures), under sedation with incremental doses of propofol, on demand and per typical institutional practices. Olympus endoscopes [GF-UCT140-AL5 and GF-UCT180 + EU-ME2 Premier Plus (EVIS EUS) (Olympus Optical Co. Europa, Hamburg, Germany)] were used in all cases. After the splenic artery was located, a small turn clockwise was performed, while slightly pulling the scope; this procedure helped the examiner to depict the pancreatic body and then the pancreatic tail up to the splenic hilum. For discrimination between the body and tail, lesions located more proximal to the hilum were deemed to represent the area of the tail. Location, size, echogenicity, margin and shape of each lesion were recorded and lesions situated in the head of the pancreas were typically approached transduodenally and those in the body/tail by a transgastric approach. All FNAs were conducted in a standardized manner, as previously described [7]. Briefly, the lesion of interest, including the local regional vasculature, was evaluated by applying the color Doppler function to determine the best suitable site for puncture. Upon establishment of a vessel-free tract, a 22- or 25-gauge needle (EZ Shot 2, and after September 2018 when it became commercially available, EZ Shot 3, Olympus Optical Co. Europa, Hamburg, Germany) was advanced through the working channel of the endoscope and finally inserted into the lesion under constant ultrasonographic guidance. When inside the lesion, the stylet was removed, and material was aspirated by continuous gentle suction into a prefixed 10 mL suction syringe. Once the aspiration was complete, the needle was withdrawn along with removal of the catheter system through the biopsy channel, thus designating a needle pass. Each needle pass consisted of 9–12 needle strokes aiming at 3–4 different positions in the lesion. The acquired material was inspected by the endoscopist after being placed in the cytology preservative and the number of additional passes in each case was based on the endoscopist’s macroscopic assessment. The decision to finish each EUS-FNA was made when the sample was considered to be adequate to produce a diagnosis, or if the needle displayed technical malfunction (e.g., due to bending). On-site cytopathologic assessment was not available in any case.

### 2.4. Cytologic Analysis

Following aspiration, the needle was withdrawn from the endoscope, washed in CellSolutions Red Lytic General Cytology Preservative (CELLSOLUTIONS GmBH, Wasserburg, Germany) and sent immediately to the cytopathology department for analysis by experienced gastrointestinal pathologists. In specimens in which tissue micro-fragments were visualized, the whole specimen was filtered through a nylon mesh tissue bag (Fisherbrand Nylon Biopsy Bags, Thermo Fisher Scientific) in order to prepare a cell-block and fixed immediately in 10% neutral buffered formalin. The remaining fluid was centrifuged and processed for liquid-based cytology (ThinPrep 2000 Processor, Hologic Inc. Marlborough MA). Specimens with no visible micro-fragments were processed for liquid-based cytology (LBC) only. At least 2 LBC smears were prepared from each specimen. In specimens with very low cellularity, we used the remaining fluid to prepare cytospins. All immunohistochemical stains were performed in the cell-block material. When a cell-block was not available, we performed them in the LBC or cytospin smears with results comparable to the cell-block results.

### 2.5. Study Definitions

**Figure 1:**
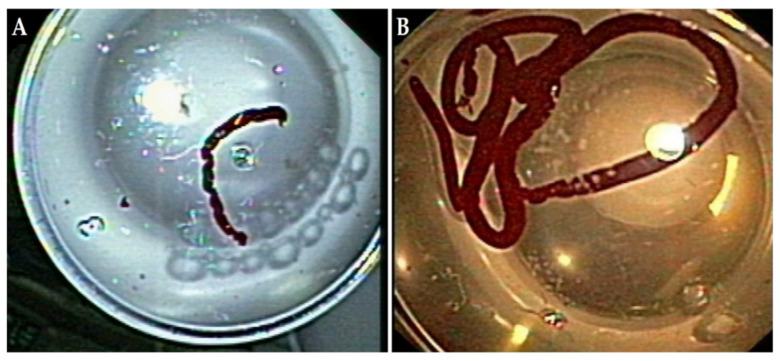
Cytology specimen after a puncture of a mass in the pancreatic body. The specimen was considered (**A**) inadequate and (**B**) adequate by the examiner. Photos are from author’s personal archive.

For the purposes of our study, overall diagnostic yield was defined as the percentage of the lesions sampled for which a tissue diagnosis was obtained (benign or malignant), whereas specimen adequacy was defined as the percentage of the lesions sampled in which the obtained material was representative of the target site and sufficient for diagnosis (Figure 1 depicts cytology specimen considered (A) inadequate and (B) adequate by the examiner). Moreover, we measured the diagnostic yield for pancreatic adenocarcinoma, which was defined as the percentage of the lesions sampled for which the diagnosis of adenocarcinoma was made [12,13]. Cytology was considered positive only if it was unequivocally diagnostic of the lesion’s origin. Specimens not containing typical cells or those with insufficient material to make a definite diagnosis were deemed as inadequate [7].

### 2.6. Study Endpoints

The primary study endpoint was to compare the overall diagnostic yield by EUS-FNA between 2 and 3 passes. Secondary endpoints comprised: (i) comparison between 2 and 3 passes regarding diagnostic yield for pancreatic adenocarcinoma (percentage of patients with positive diagnosis for pancreatic adenocarcinoma); (ii) comparison between 2 and 3 passes regarding specimen adequacy; (iii) identification of factors associated with material adequacy.

### 2.7. Statistical Analyses

The distribution of quantitative data was evaluated for normality using the Kolmogorov–Smirnov test and expressed as the mean (±SD) or median (IQR) according to their distribution. Categorical variables are shown as counts (%) and proportions are presented with the respective 95% binomial CIs. Student’s t test was used for comparisons of variables with normal distribution and a 2-sided Chi-squared test, corrected by Fisher’s exact test when appropriate, was used for comparisons of categorical variables. For all endpoints we used the odds ratio (OR) with the respective 95% confidence intervals (CI) to estimate the effect of the intervention in the two groups (two vs. three passes). Bivariate logistic regression analysis was applied to identify factors associated with overall diagnostic yield, diagnostic yield for adenocarcinoma and specimen adequacy (dependent variables) and the unadjusted ORs (95% CI) are presented. The model’s independent variables comprised patients’ basic demographic characteristics (gender, age), the lesion’s characteristics (size in mm and location (head/body/tail)) potentially affecting the procedure’s outcome, and the factors that have been shown in the available literature to potentially relate to EUS tissue sampling of solid pancreatic masses (needle type and number of passes in a single step—standard regression analysis) [4,6], while in the multivariable model all variables were included. A *p*-value < 0.05 was considered significant. All statistical analyses were performed with SPSS version 25 (IBM, Chicago, IL, USA).

### 2.8. Ethical Approval

The study protocol was reviewed and approved by the local Institutional Review Board (“Attikon” University General Hospital, Protocol Nr: 227/14.04.2021, decision: 4th meeting/24 April 2021). The study was conducted in accordance with the ethical principles of the Declaration of Helsinki and in compliance with good clinical practice. All patients provided written informed consent before undergoing the procedure.

## 3. Results

**Figure 2:**
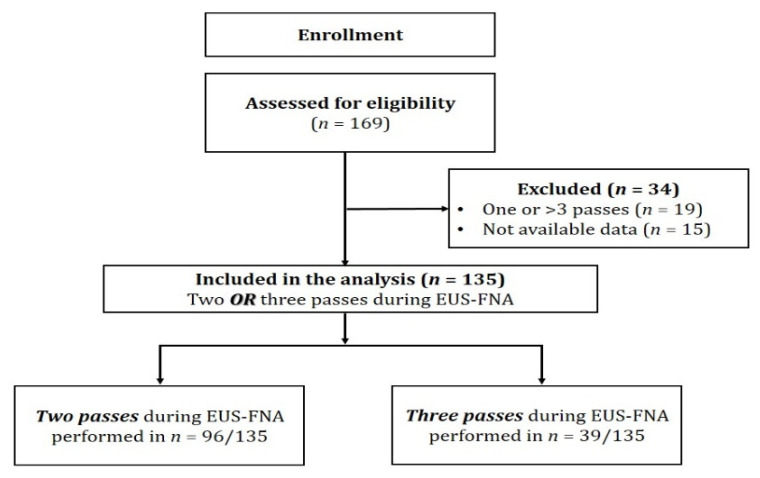
Study flowchart.

One hundred sixty-nine patients were initially assessed for eligibility, but only 135 patients (each with a single lesion) met the inclusion criteria and were finally included in the analysis (Figure 2); 49 patients (36.3%) were enrolled in Center 1 and 86 (63.7%) in Center 2.

Patients’ baseline and procedural characteristics are presented in Table 1. The majority of the lesions were located in the head of the pancreas (*n* = 101/135, 74.8%). For most EUS-FNAs, a 22G-needle was used (*n* = 120/135, 88.9%). With regard to the diagnosed malignancies (*n* = 110/135, 81.5%), pancreatic adenocarcinoma was the most frequent (*n* = 106/110, 96.4%) whereas the rest of the diagnosed malignancies included 1 case of metastatic lung cancer, 1 case of gastrointestinal stromal tumor and 2 cases of mucinous neoplasms with low- and high-grade dysplasia, respectively; 96 patients underwent 2 needle passes, whereas 39 patients underwent 3 passes; no difference was noted between these two patient groups regarding clinical or procedural characteristics. The overall diagnostic yield, diagnostic yield for adenocarcinoma and specimen adequacy were 90.4% (95% CI: 84.1–94.8), 78.5% (95% CI: 70.6–85.1) and 83.7% (95% CI: 76.4–89.5), respectively (Table 1).

### 3.1. Endpoints

#### 3.1.1. Primary Endpoint

**Figure 3:**
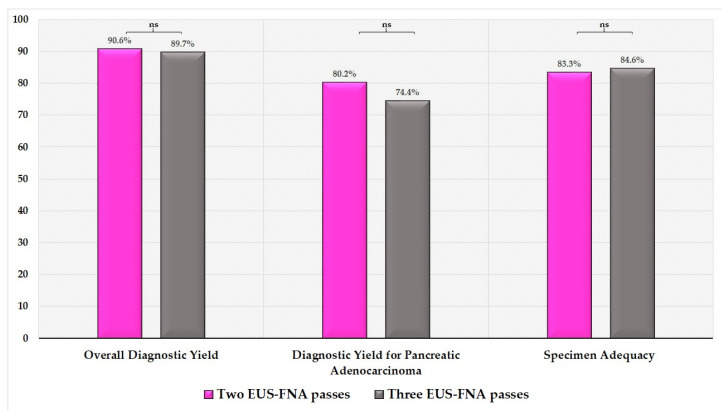
Overall diagnostic yield, diagnostic yield for pancreatic adenocarcinoma and specimen adequacy for 2 and 3 EUS-FNA passes (ns = not significant).

No statistically significant difference regarding the overall diagnostic yield was detected between patients undergoing 2 compared to 3 needle passes (87/96 (90.6%; 95% CI: 82.9–95.6) vs. 35/39 (89.7%; 95% CI: 75.8–97.1); OR (95% CI): 1.10 (0.31–3.82); *p* = 0.87, Figure 3).

#### 3.1.2. Secondary Endpoints

1. Similarly, no statistically significant difference in terms of diagnostic yield for adenocarcinoma between patients undergoing 2 passes compared to those with 3 passes was detected (77/96 (80.2%; 95% CI: 70.5–87.6) vs. 29/39 (74.4%; 95% CI: 57.9–82.9); OR (95% CI): 1.39 (0.58–3.35); *p* = 0.45, Figure 3).

2. No statistically significant difference between the two groups regarding specimen adequacy was found (80/96 (83.3%; 95% CI: 74.4–90.2) vs. 33/39 (84.6%; 95% CI: 69.5–94.1); OR (95% CI): 0.90 (0.32–2.52); *p* = 0.86, Figure 3).

3. We then analyzed possible associations of various parameters with overall diagnostic yield, specimen adequacy and diagnostic yield for adenocarcinoma. Patients in whom a tissue diagnosis was obtained (overall diagnostic yield) or adenocarcinoma was diagnosed (diagnostic yield for adenocarcinoma) were older than those without a definite tissue diagnosis or diagnosis of adenocarcinoma, respectively (67.9 ± 11.6 versus 60.4 ± 17.0 years, *p* = 0.024 and 68.2 ± 12.0 versus 62.3 ± 14.5 years, *p* = 0.041, respectively). The proportion of lesions located in the body of the pancreas for which specimen adequacy was present was significantly higher (21.1% vs. 3.8%, *p* = 0.029). This was also the case for diagnosis of adenocarcinoma (21.6% vs. 6.1%, p=0.027). Ιn the multivariate analysis, overall diagnostic yield, specimen adequacy and diagnostic yield for adenocarcinoma were independent of age and location of the lesion, as demonstrated in Table 2. Similarly, no association was found for gender, size of the lesion, needle type, and the number of passes (three versus two) in regard to all three outcome measures.

## 4. Discussion

Current guidelines recommend 3 to 4 needle passes to obtain the optimal tissue sample for a diagnosis in patients with solid pancreatic masses, in the absence of on-site cytologic evaluation, which is the usual case in most endoscopic units [6]. However, the abovementioned recommendation is based on low-quality evidence, due to the fact that data on this topic still remain scarce. In addition, the incremental benefit of additional number of passes, that is, beyond 4, has also been a matter of debate [7,9,14]. Hence, this large, retrospective study went a step further for the first time, providing the insight that equivalent accuracy can be achieved even with 2 passes during EUS-FNA.

Regarding our primary endpoint, our analysis suggests that 2 needle passes during EUS-FNA of solid pancreatic masses have the potential to provide an equally diagnostic tissue sample, as compared to 3 passes. This finding seems to be corroborated by similar references in the literature that also seem to suggest that perhaps for EUS-FNA “less is indeed more” from the point of one needle pass and on [12,14], with data suggesting that a cytological diagnosis can even be established by the first needle pass in many cases (75%) [9]. A potential explanation for these findings could be due to the improvement in the individual endoscopist’s performance, since the examination not only remains a technically demanding procedure with a slow learning curve but also with a high “operator-dependence” and significant interoperator variability [15,16]. A few years ago, gross visual assessment of specimen adequacy during EUS-guided FNA of pancreatic masses was found to be ambiguous, as neither trained EUS technologists nor cytotechnologists were able to reliably evaluate it [17]. In this regard, the endosonographer’s level of expertise and technique has a pivotal role in the examination’s diagnostic accuracy, and as has evolved over time, nowadays even 2 passes might suffice to diagnose the nature of a solid pancreatic lesion [18,19]. Similarly, the optimization of existing technological modalities, i.e., new echoendoscopes as well as the incorporation of novel sophisticated devices, i.e., needle types and acquisition techniques (i.e., fanning, slow capillary pull-through, wet suction) could also account for “narrowing the gap” regarding the value of additional passes [20,21].

According to the EUS-FNA protocol technique used in both centers, the decision to conduct a 3rd pass is based on the macroscopic assessment of the acquired material by the endoscopist, meaning that if after 2 passes the material is macroscopically sensed to be inadequate, a 3rd pass is then performed. Taking into account the similar diagnostic accuracy of 3 passes compared to 2, one might conclude that a 3rd pass probably cannot offer any additional diagnostic benefit when 2 passes have already “failed”. This can be partially explained, at least in the case of pancreatic adenocarcinoma, by the fact that this type of tumor is often fibrotic due to a prominent desmoplastic/stromal reaction, which makes sample acquisition challenging per se. Although macroscopic evaluation of the obtained specimen has been associated with higher diagnostic performance in the histologic and cytologic examinations only with the use of 19-gauge needles [22], the results of our study might imply that macroscopic evaluation can also be successfully applied on samples from 22- and 25-gauge needles when combined with the assessment of the samples by experienced and dedicated cytologists and/or pathologists.

Another point worth mentioning is the fact that 3 needle passes failed to provide an incremental benefit compared to 2 in cases that were in their majority pancreatic adenocarcinomas; this may be linked to inherent biological properties of the tumor itself, e.g., its rapid growth rate, which results in relatively large lesion dimensions, as data suggests that the sensitivity and diagnostic accuracy of EUS-FNA are not only higher for lesions ≥ 10 mm, but also that they correlate with mass size [23,24]. A large lesion not only provides a wider visualization field and facilitates accurate targeting but may also allow a longer needle stroke; thus, acquisition of larger volume samples is possible and displacement of the target lesion induced by advancement of the needle is avoided [9]. Finally, the advent of modern, highly sensitive cytological methods may also enable a firm diagnosis of malignancy to be made, despite lower volume samples [25,26].

Establishing a diagnosis remains the cornerstone of EUS-FNA [27,28]. However, there are also several aspects of the procedure that may be underestimated that still have significant implications in everyday clinical practice. Performing additional FNA passes prolongs the procedure time; thus, exposing patients to higher potential for procedure or sedation-related adverse events [29]. This is important as patients undergoing these procedures are usually of advanced age, or suffer from comorbidities that make them particularly susceptible to the occurrence of adverse events. Moreover, one should bear in mind that the accumulation of proteinaceous material or blood within the needle or trocar after each additional pass may damage the needle beyond redemption [30,31]. In this sense, performing as fewer passes as possible abolishes the need for additional needles, which translates into a smaller financial burden [11]. Although we did not perform an official cost-effectiveness analysis, our results are in this direction.

Our multivariate analysis failed to reveal any correlation between lesion- or procedure-related factors and diagnostic accuracy; however, this issue remains ambiguous, with concurrent studies reporting conflicting results. No significant relationship between lesion size and location, or needle size and diagnostic yield was reported in similar reports [12,32]; the operator’s experience was the only significant predictor of accuracy in another study [18], while lesion size of 15 mm or less, location of the target lesion in the pancreatic head and the presence of a neuroendocrine tumor (NET) were reported to require 2 passes elsewhere [9]. As far as EUS-guided tissue sampling is considered, a recent network meta-analysis demonstrated that no specific technique was superior with regard to diagnostic accuracy, sample adequacy, or histologic procurement rate [4]. Beyond a potential type II statistical error—since our study was not powered to address this—our findings may also reflect the homogeneity of our population. Enrollment exclusively of patients with solid pancreatic lesions is associated with a higher pretest probability for malignancy.

The strengths of this study are the use of a standardized EUS-FNA protocol technique across both centers. Application of stringent diagnostic criteria and inclusion only of patients with solid pancreatic lesions should be included in the study’s assets as well. Finally, inclusion of 22- and 25-gauge needles accurately reflects real-world everyday clinical practice.

On the other hand, there are also limitations that merit attention. The study’s principal drawback is its retrospective design. Second, the performance of EUS-FNA procedures by expert endosonographers across tertiary centers may limit the generalizability of the results. Third, one might refute our effort to identify factors that may influence the performance of the examination given that this should be ideally addressed within prospectively conducted studies. Another point that could fuel some dispute is the fact that FNA needles were used instead of fine-needle biopsy (FNB) ones; in fact, ESGE-guidelines recommend use of either FNA or FNB needles for routine sampling of solid masses, except when the goal is to obtain a core tissue specimen, where 22-gauge FNB needles or larger FNA needles (19-gauge) should be preferred [6]. Our decision to use “standard” (22- and 25-gauge) FNA needles, was based on various reasons, including the fact that the relevant recommendation is a weak one that is based on low-quality evidence, as well as our previous work, where we demonstrated that even 22-gauge FNA needles can suffice to obtain a core specimen [7]. Moreover, our previous findings were supported by our recent network meta-analysis that failed to display any significant difference deriving from a special type of needle [4]. The high yield of core specimens that we managed to obtain in our present study seems to confirm our previous findings and support this rationale. Needless to say, the significantly lower cost of FNA compared to FNB needles was also a major contributing factor in making the decision to use this needle type. Another limitation is that the number of additional passes in each case was based on each physician’s macroscopic assessment. However, in the absence of ROSE, the macroscopic assessment does not guarantee the adequacy of the sample. Thus, the clinical question of the study remains valid. The unavailability of ROSE, despite the fact that both centers were tertiary, might also attract criticism; however, this is the case in many endoscopic departments due to limited financial resources and personnel shortages and as such our study accurately replicates everyday clinical practice conditions. Finally, one could also ask why we compared 2 vs. 3 passes when alternatively, we could compare all passes independently to check for differences between different pass numbers (i.e., 1 vs. 2 vs. 3 vs. 4, etc.). This however was outside the scope of our study, which was to test the lower limit that was set in the ESGE guidelines and to assess if even fewer passes than this cutoff would suffice to make a definite diagnosis.

## 5. Conclusions

To conclude, our study provides evidence that 2 needle passes lead to a similar diagnostic yield compared to 3 passes during EUS-FNA of solid pancreatic masses. However, these results should be interpreted with caution, as endosonographers evaluated the specimens and decided whether to stop or continue the EUS-FNA procedure. While further prospectively collected data are warranted to determine the optimal number of passes in EUS-FNA for a solid pancreatic mass, the experienced endoscopist may decide to perform only 2 passes, especially when malignancy is strongly suspected, on a case-by-case basis.

## Figures and Tables

**Table 1:** Patient’ baseline clinical and procedural characteristics.

	All Patients (*n* = 135)	2 Passes (*n* = 96)	3 Passes (*n* = 39)	*p* *
*Patient characteristics*				
Gender (males)	83 (61.5%)	55 (57.3%)	28 (71.8%)	0.125
Age (years) ^#^	66.9 ± 12.8	67.6 ± 13.3	65.2 ± 11.2	0.332
Lesion location (head/body/tail)	101 (74.8%)/24 (17.8%)/10 (7.4%)	71 (74%)/19 (19.8%)/6 (6.3%)	30 (76.9%)/5 (12.8%)/4 (10.3%)	0.480
Size of lesion (mm) ^#^	28.8 ± 11.9	27.9 ± 12.7	31.2 ± 9.7	0.115
*Procedural characteristics*				
Needle type (22G)	120 (88.9%)	85 (88.5%)	35 (89.7%)	1.000
Procedure time, mean (SD), minutes	23.2 (8.7)	22.2 (9.2)	24.1 (8.1)	0.19
Propofol dose (mg)	204.7 ± 88.7	188.7 ± 75.4	223.0 ± 100.7	0.210
Overall diagnostic yield	122 (90.4%)			
Diagnostic yield for adenocarcinoma	106 (78.5%)			
Specimen adequacy	113 (83.7%)			

* *p* value for comparisons between 2 and 3 passes; ^#^ expressed as mean ± standard deviation; G: gauge.

**Table 2:** Multivariate analysis of factors associated with overall diagnostic yield, diagnostic yield for adenocarcinoma and specimen adequacy. OR, 95%CI: odds ratio with 95% confidence intervals.

Parameters	Overall Diagnostic Yield (OR, 95%CI)	*p* Value	Diagnostic Yield for Adenocarcinoma (OR, 95%CI)	*p* Value	Specimen Adequacy (OR, 95%CI)	*p* Value
Age (years)	1.043 (0.999–1.089)	0.063	1.033 (0.998–1.069)	0.092	1.031 (0.994–1.069)	0.056
Gender (Female vs. Male)	0.958 (0.275–3.342)	0.844	1.117 (0.440–2.833)	0.374	1.463 (0.521–4.108)	0.687
Lesion size	1.010 (0.954–1.069)	0.569	0.994 (0.954–1.035)	0.591	1.008 (0.964–1.055)	0.918
Size (<20 mm vs. ≥20 mm)	2.010 (0.254–11.069)	0.419	2.214 (0.204–12.031)	0.119	1.408 (0.104–11.05)	0.180
Lesion location (Body vs. Head)	2.730 (0.346–23.599)	0.195	3.156 (0.667–14.945)	0.163	5.610 (0.682–46.126)	0.179
Lesion location (Tail vs. Head)	0.275 (0.056–1.355)	0.302	0.293 (0.072–1.189)	0.275	0.328 (0.076–1.408)	0.174
Number of passes (3 vs. 2)	1.614 (0.178–14.601)	0.736	1.029 (0.389–2.726)	0.827	1.423 (0.473–4.285)	0.767
Needle type (25G vs. 22G)	2.243 (0.260–19.341)	0.463	4.075 (0.495–3.531)	0.205	3.126 (0.371–26.322)	0.134

## Data Availability

The data presented in this study are available on request from the corresponding author.

## References

[1] Papanikolaou I.S. (2020). Quality in pancreatic endoscopic ultrasound: What’s new in 2020?. Ann. Gastroenterol..

[2] Matsubayashi H., Matsui T., Yabuuchi Y., Imai K., Tanaka M., Kakushima N., Sasaki K., Ono H. (2016). Endoscopic ultrasonography guided-fine needle aspiration for the diagnosis of solid pancreaticobiliary lesions: Clinical aspects to improve the diagnosis. World J. Gastroenterol..

[3] Hewitt M.J., McPhail M.J., Possamai L., Dhar A., Vlavianos P., Monahan K.J. (2012). EUS-guided FNA for diagnosis of solid pancreatic neoplasms: A meta-analysis. Gastrointest. Endosc..

[4] Facciorusso A., Wani S., Triantafyllou K., Tziatzios G., Cannizzaro R., Muscatiello N., Singh S. (2019). Comparative accuracy of needle sizes and designs for EUS tissue sampling of solid pancreatic masses: A network meta-analysis. Gastrointest. Endosc..

[5] Iglesias-Garcia J., Dominguez-Munoz J.E., Abdulkader I., Larino-Noia J., Eugenyeva E., Lozano-Leon A., Forteza-Vila J. (2011). Influence of On-Site Cytopathology Evaluation on the Diagnostic Accuracy of Endoscopic Ultrasound-Guided Fine Needle Aspiration (EUS-FNA) of Solid Pancreatic Masses. Am. J. Gastroenterol..

[6] Polkowski M., Jenssen C.C., Kaye P.V., Carrara S., Deprez P., Ginès A., Fernández-Esparrach G.G., Eisendrath P., Aithal G.P., Arcidiacono P.P. (2017). Technical aspects of endoscopic ultrasound (EUS)-guided sampling in gastroenterology: European Society of Gastrointestinal Endoscopy (ESGE) Technical Guideline – March 2017. Endoscopy.

[7] Möller K., Papanikolaou I.S., Toermer T., Delicha E.M., Sarbia M., Schenck U., Koch M., Al-Abadi H., Meining A., Schmidt H. (2009). EUS-guided FNA of solid pancreatic masses: High yield of 2 passes with combined histologic-cytologic analysis. Gastrointest. Endosc..

[8] Suzuki R., Irisawa A., Bhutani M.S., Hikichi T., Takagi T., Sato A., Sato M., Ikeda T., Watanabe K., Nakamura J. (2012). Prospective evaluation of the optimal number of 25-gauge needle passes for endoscopic ultrasound-guided fine-needle aspiration biopsy of solid pancreatic lesions in the absence of an onsite cytopathologist. Dig. Endosc..

[9] Uehara H., Sueyoshi H., Takada R., Fukutake N., Katayama K., Ashida R., Ioka T., Takenaka A., Nagata S., Tomita Y. (2015). Optimal number of needle passes in endoscopic ultrasound-guided fine needle aspiration for pancreatic lesions. Pancreatol..

[10] Cherian P.T., Mohan P., Douiri A., Taniere P., Hejmadi R.K., Mahon B.S. (2010). Role of endoscopic ultrasound-guided fine-needle aspiration in the diagnosis of solid pancreatic and peripancreatic lesions: Is onsite cytopathology necessary?. HPB.

[11] Erickson R.A., Sayage-Rabie L., Beissner R. (2000). Factors predicting the number of EUS-guided fine-needle passes for diagnosis of pancreatic malignancies. Gastrointest. Endosc..

[12] Ge P.S., Wani S., Watson R.R., Sedarat A., Kim S., Marshall C., Wilson R.H., Makker J., Mohamadnejad M., Komanduri S. (2018). Per-Pass Performance Characteristics of Endoscopic Ultrasound-Guided Fine-Needle Aspiration of Malignant Solid Pancreatic Masses in a Large Multicenter Cohort. Pancreas.

[13] Wani S., Muthusamy V.R., Komanduri S. (2014). EUS-guided tissue acquisition: An evidence-based approach (with videos). Gastrointest. Endosc..

[14] Mohamadnejad M., Mullady D., Early D.S., Collins B., Marshall C., Sams S., Yen R., Rizeq M., Romanas M., Nawaz S. (2017). Increasing Number of Passes Beyond 4 Does Not Increase Sensitivity of Detection of Pancreatic Malignancy by Endoscopic Ultrasound–Guided Fine-Needle Aspiration. Clin. Gastroenterol. Hepatol..

[15] Eloubeidi M.A., Tamhane A. (2005). EUS-guided FNA of solid pancreatic masses: A learning curve with 300 consecutive procedures. Gastrointest. Endosc..

[16] Wani S., Coté G.A., Keswani R., Mullady D., Azar R., Murad F., Edmundowicz S., Komanduri S., McHenry L., Al-Haddad M.A. (2013). Learning curves for EUS by using cumulative sum analysis: Implications for American Society for Gastrointestinal Endoscopy recommendations for training. Gastrointest. Endosc..

[17] Nguyen Y.P., Maple J.T., Zhang Q., Ylagan L.R., Zhai J., Kohlmeier C., Jonnalagadda S., Early D.S., Edmundowicz S.A., Azar R.R. (2009). Reliability of gross visual assessment of specimen adequacy during EUS-guided FNA of pancreatic masses. Gastrointest. Endosc..

[18] Harewood G.C., Wiersema L.M., Halling A.C., Keeney G.L., Salamao D.R., Wiersema M.J. (2002). Influence of EUS training and pathology interpretation on accuracy of EUS-guided fine needle aspiration of pancreatic masses. Gastrointest. Endosc..

[19] Hupp M.M., Khan S., Dincer H.E., Mallery J.S., Shyne M.T., Mettler T., Stewart J., Amin K. (2021). Evaluation and Comparison of Performance Parameters and Impact of Telepathology and Operator Experience on Endobronchial and Endoscopic Ultrasound-Guided Fine-Needle Aspiration. Am. J. Clin. Pathol..

[20] Weston B.R., Bhutani M.S. (2013). Optimizing Diagnostic Yield for EUS-Guided Sampling of Solid Pancreatic Lesions: A Technical Review. Gastroenterol. Hepatol..

[21] Irisawa A., Yamabe A., Bhutani M.S., Shibukawa G., Fujisawa M., Sato A., Yoshida Y., Arakawa N., Ikeda T., Igarashi R. (2016). Efforts to improve the diagnostic accuracy of endoscopic ultrasound-guided fine-needle aspiration for pancreatic tumors. Endosc. Ultrasound.

[22] Iwashita T., Yasuda I., Mukai T., Doi S., Nakashima M., Uemura S., Mabuchi M., Shimizu M., Hatano Y., Hara A. (2015). Macroscopic on-site quality evaluation of biopsy specimens to improve the diagnostic accuracy during EUS-guided FNA using a 19-gauge needle for solid lesions: A single-center prospective pilot study (MOSE study). Gastrointest. Endosc..

[23] Park C., Kim H.J., Kim S.Y., Lee S.S., Byun J.H., Kim S.C., Kim M.-H. (2019). Growth rate of serous pancreatic neoplasms in vivo: A retrospective, observational study. Acta Radiol..

[24] Sugiura R., Kuwatani M., Hirata K., Sano I., Kato S., Kawakubo K., Sakamoto N. (2019). Effect of Pancreatic Mass Size on Clinical Outcomes of Endoscopic Ultrasound-Guided Fine-Needle Aspiration. Dig. Dis. Sci..

[25] Crinò S.F., Larghi A., Bernardoni L., Parisi A., Frulloni L., Gabbrielli A., Parcesepe P., Scarpa A., Manfrin E. (2019). Touch imprint cytology on endoscopic ultrasound fine-needle biopsy provides comparable sample quality and diagnostic yield to standard endoscopic ultrasound fine-needle aspiration specimens in the evaluation of solid pancreatic lesions. Cytopathology.

[26] Pouliakis A., Karakitsou E., Margari N., Bountris P., Haritou M., Panayiotides J., Koutsouris D., Karakitsos P. (2016). Artificial Neural Networks as Decision Support Tools in Cytopathology: Past, Present, and Future. Biomed. Eng. Comput. Biol..

[27] Pouw R.E., Barret M., Biermann K., Bisschops R., Czakó L., Gecse K.B., de Hertogh G., Hucl T., Iacucci M., Jansen M. (2021). Endoscopic tissue sampling – Part 1: Upper gastrointestinal and hepatopancreatobiliary tracts. European Society of Gastrointestinal Endoscopy (ESGE) Guideline. Endosc..

[28] Wani S., Wallace M.B., Cohen J., Pike I.M., Adler D.G., Kochman M.L., Lieb J.G., Park W.G., Rizk M.K., Sawhney M.S. (2015). Quality indicators for EUS. Gastrointest. Endosc..

[29] McQuaid K.R., Laine L. (2008). A systematic review and meta-analysis of randomized, controlled trials of moderate sedation for routine endoscopic procedures. Gastrointest. Endosc..

[30] Erickson R.A., Sayage-Rabie L., Avots-Avotins A. (1997). Clinical utility of endoscopic ultrasound-guided fine needle aspiration. Acta Cytol..

[31] A Erickson R., Tretjak Z. (2000). Clinical Utility of Endoscopic Ultrasound and Endoscopic Ultrasound-Guided Fine Needle Aspiration in Retroperitoneal Neoplasms. Am. J. Gastroenterol..

[32] Uehara H., Ikezawa K., Kawada N., Fukutake N., Katayama K., Takakura R., Takano Y., Ishikawa O., Takenaka A. (2011). Diagnostic accuracy of endoscopic ultrasound-guided fine needle aspiration for suspected pancreatic malignancy in relation to the size of lesions. J. Gastroenterol. Hepatol..

